# Factors Associated with Condomless Anal Sex and Absence of Pre-Exposure Prophylaxis (PrEP) Use Among Brazilian Men Who Have Sex with Men: A Cross-Sectional Study

**DOI:** 10.3390/idr17060149

**Published:** 2025-12-12

**Authors:** Laelson Rochelle Milanês Sousa, Patrícia Thais Cardoso da Silva, Allan Araujo Rodrigues, Márcio José dos Santos Silva, José Carlos Vinícius Jansen de Paz, Breno da Silva Oliveira, Daniel de Macêdo Rocha, Maria Wiklander, Elucir Gir, Renata Karina Reis

**Affiliations:** 1 Nursing Course, State University of Maranhão, Coroata 65665-000, Brazil; patriciathais12@hotmail.com (P.T.C.d.S.); rodriguesallan728@gmail.com (A.A.R.); marciosantosj2@gmail.com (M.J.d.S.S.); viniciusjansen11@gmail.com (J.C.V.J.d.P.); 2 Department of Nursing, Federal University of Mato Grosso do Sul, Coxim 79400-000, Brazil; oliveira_breno@ufms.br (B.d.S.O.); daniel.macedo@ufms.br (D.d.M.R.); 3 Division of Nursing, Department of Neurobiology, Care Sciences and Society, Karolinska Institutet, 171-77 Stockholm, Sweden; maria.wiklander@ki.se; 4 Ribeirão Preto College of Nursing, University of São Paulo, Ribeirao Preto 14040-902, Brazil; egir@eerp.usp.br (E.G.); rkreis@eerp.usp.br (R.K.R.)

**Keywords:** HIV, sexually transmitted diseases, pre-exposure prophylaxis, men who have sex with men, sexual and gender minorities

## Abstract

Background: Men who have sex with men (MSM) in Brazil remain disproportionately affected by HIV. Combination prevention strategies, including Pre-Exposure Prophylaxis (PrEP), are critical, yet adherence remains a challenge. This study aimed to identify factors associated with the simultaneous practice of condomless anal sex and non-use of PrEP among Brazilian MSM. Methods: A national cross-sectional study was conducted in 2020 via an online questionnaire disseminated on social media and dating apps. The outcome was defined as reporting condomless anal sex and no PrEP use in the previous year. Bivariate and multivariate logistic regression analyses were performed. Results: Among 1357 MSM participants, a high proportion (69.4%) reported condomless anal sex without PrEP use. Factors significantly associated with this behavior included being younger (18–28 years; AOR: 2.59), identifying as homosexual (AOR: 6.04), bisexual (AOR: 5.30), or pansexual (AOR: 8.67), having a steady partner (AOR: 4.57), engaging primarily in receptive or insertive anal sex, and having a prior STI diagnosis (AOR: 1.49). Conclusions: The confluence of condomless sex and PrEP non-use reveals a significant vulnerability profile among young MSM in Brazil, even within steady relationships. These findings highlight the originality of examining this combined behavioral outcome and underscore the urgent need for targeted, culturally sensitive prevention strategies that address risk perception and enhance PrEP uptake to meet the UNAIDS 2030 goals.

## 1. Introduction

Condomless anal sex and the absence of Pre-Exposure Prophylaxis (PrEP) among men who have sex with men (MSM) are high-risk behaviors for HIV acquisition and transmission. In this context, combination prevention has emerged as a strategy that integrates different methods to prevent HIV: biomedical, behavioral, and structural approaches [1,2]. Consistent use of external condoms and PrEP is recommended for key populations like MSM; however, it is necessary to analyze adherence conditions to these strategies [1].

In Brazil, PrEP has been available through the public health system (Sistema Único de Saúde—SUS) since 2017, primarily targeting key populations, including MSM. Despite its availability, uptake has been slower than expected, and significant regional disparities in access persist. Structural and psychosocial barriers, such as stigma in healthcare services, low risk perception, and insufficient knowledge about PrEP, remain major challenges to its effective implementation [3,4]. Therefore, understanding the factors that lead MSM to not use PrEP while simultaneously engaging in condomless anal sex is a public health priority.

HIV infection remains a significant public health challenge globally, despite significant advances in antiretroviral treatment and combined prevention strategies. It is estimated that in 2024 alone, approximately 630,000 people died as a result of AIDS-related illnesses, a considerably lower number than in 2004, when there were about 2.1 million deaths. Even with these reductions, AIDS mortality remains higher than the UNAIDS target for 2025, which is to reduce this total to less than 250,000 deaths. The prevalence of HIV in the general Brazilian population is approximately 0.49%. However, 53.6% of HIV cases reported in Brazil in 2023 occurred among MSM [5].

Brazilian MSM face stigma, discrimination, and marginalization, which create barriers to accessing healthcare services, impacting HIV testing and the adoption of other prevention methods [6], such as consistent male condom use [1]. Low adherence to combination prevention remains a significant challenge to achieving the global goals proposed by UNAIDS for controlling the HIV epidemic by 2030 [7].

International studies have identified factors related to low adherence to combination prevention. In a randomized clinical trial conducted in France and Canada, condomless anal sex and no PrEP use among MSM were influenced by the following factors: a depressive episode in the last 12 months; a higher number of sexual encounters in the previous four weeks; and sexual intercourse under the influence of alcohol [8]. Another study conducted in 18 Latin American countries, involving 53,166 MSM, found that condomless anal sex was associated with commercial sex work, multiple drug use, homophobic abuse, and alcohol dependence [9,10].

Other factors related to condomless anal sex may involve individual and behavioral barriers such as physical discomfort, limited knowledge about HIV/STIs, and the use of psychoactive substances. Environmental and structural factors, including the inaccessibility of condoms and power imbalances in sexual relationships, have also been reported [11]. While international studies have identified factors for low adherence to individual prevention methods [12], the Brazilian context presents unique socio-cultural dynamics. Previous national studies have often focused on either condom use or PrEP uptake separately. However, the simultaneous occurrence of condomless anal sex and PrEP non-use represents a distinct and higher-risk behavioral profile that is not yet fully understood [1]. These findings underscore the need to investigate the determinants of this combined outcome to inform more effective, integrated health strategies. This study, therefore, aims to identify the factors associated with condomless anal sex and the absence of PrEP use among Brazilian MSM.

## 2. Materials and Methods

### 2.1. Study Design, Participants, and Data Collection

This was a cross-sectional, analytical study conducted with men who have sex with men (MSM) from all regions of Brazil. The inclusion criteria were being 18 years or older, identifying as male (self-identification as a man-gender identity), being a Brazilian national, having access to the internet, and having had at least one sexual encounter with another man in their lifetime. The exclusion criterion was failure to complete the data collection questionnaire.

Data were collected electronically between April and May 2020 using a questionnaire developed and hosted on the SurveyMonkey platform. Various social media strategies were employed to recruit participants. Initially, the survey link containing the questionnaire was shared on Instagram through pre-established partnerships between the research team and Brazilian digital influencers, who distributed the survey to their followers. After mass dissemination on Instagram, other social media platforms were used to recruit participants: Facebook, Twitter, WhatsApp, and Telegram groups. The link to the questionnaire was accompanied by an informational message asking participants to share it with other MSM, thereby reaching individuals who were not in the online environments where the link was initially posted. Additionally, the link was shared on MSM dating apps, such as Grindr.

In this study, convenience sampling was used to collect online data. A total of 1830 MSM accessed the questionnaire. However, 473 MSM did not complete the questionnaire or failed to answer the question regarding anal sex practice. After applying the inclusion and exclusion criteria, a final sample of 1357 MSM was obtained.

### 2.2. Questionnaire and Variables

The data collection questionnaire was divided into three thematic blocks with the following organization: the first block contained sociodemographic variables (age range; gender identity; sexual orientation; years of education; self-declared skin color; employment status; marital status; sex worker status; region of Brazil); the second block contained behavioral variables (alcohol use; tobacco use; steady sexual partner; number of sexual partners in the last year; most frequent sexual practice; condomless anal sex in the past year; consistent condom use in all sexual encounters; use of a male condom during the last sexual encounter; use of lubricants during sexual intercourse); and the third block covered data on HIV and STI prevention (ever tested for HIV; knowledge of a location to obtain an HIV test; received counseling from a healthcare professional about HIV testing; received counseling from a friend about HIV testing; received free external condoms in the past 12 months; read online information about HIV prevention in the past 12 months; read printed material about prevention in the past 12 months; diagnosed with an STI). Participants accessed and signed the ethical consent form before beginning the questionnaire. The questionnaire guide used was developed by the authors specifically for this study and can be found in Supplementary Materials S1. It is important to note that the instrument was not subjected to a formal psychometric validation process, which constitutes a limitation of this study.

Condomless anal sex and no PrEP use was considered the outcome of this study. This variable was measured dichotomously (yes/no) using the question: “Did you engage in condomless anal sex and not use PrEP in the last 12 months?”, while the/all other variables in the questionnaire were used as independent variables.

### 2.3. Statistical Analysis

Statistical analyses included frequencies and percentages to describe the general characteristics of MSM. Data were analyzed using binary logistic regression to determine the effect of independent variables on the practice of condomless anal sex and no PrEP use. Independent variables with statistical significance at *p* < 0.2 [13] were included in the regression model using the “enter” method (forward). Pearson’s chi-square test was used for bivariate analysis. Odds Ratios (OR), Crude Odds Ratio (COR), and Adjusted Odds Ratio (AOR) were calculated, along with 95% Confidence Intervals and a significance level of 5% (α = 0.05), using the Jamovi software (version 2.3, The jamovi Project, Sydney, Australia). The ROC curve was used to evaluate the overall predictive performance and discriminatory power of the final multivariate model. The area under the curve (AUC) provides a global measure of the model’s ability to correctly classify individuals who engage in the risk behavior versus those who do not, based on the set of predictors identified [14].

### 2.4. Ethical Approval and Consent to Participate

This research adhered to all guidelines of the Helsinki Declaration. The study was approved by the Research Ethics Committee of the Ribeiroo Preto School of Nursing, under process 3,172,445, in accordance with the recommendations of Resolution 466/12 of the National Health Council. Participation was voluntary and conditional upon the electronic signature of the Informed Consent Form (ICF). This document outlined the research procedures, objectives, risks, and benefits, and ensured anonymity, confidentiality, privacy, and the protection of information, as well as the use of data for scientific purposes.

## 3. Results

A total of 1357 MSM (100%) participated in this study. The majority reported being white (623; 45.9%), within the age range of 18 to 28 years (1008; 73.3%), having more than 11 years of schooling (1091; 80.4%), and identifying as homosexual (1115; 82.2%). A high proportion of participants had already undergone HIV testing (1076; 79.3%) and had engaged in condomless anal sex (942; 69.4%). Additionally, a considerable portion of participants had not received free external condoms (546; 40.2%), as shown in Table 1.

The bivariate analysis indicated that a wide range of sociodemographic, behavioral, and clinical variables were associated with engaging in condomless anal sex without PrEP. Notably, factors related to sexual identity, relationship status (such as having a steady partner), substance use, inconsistent condom use, and a history of STI diagnosis showed statistically significant associations (*p* < 0.05). These initial findings suggest a multifactorial profile of vulnerability. The complete results of the bivariate analysis are detailed in Table 2.

After adjusting for confounding variables in the multivariate logistic regression model, several factors remained as independent predictors of condomless anal sex without PrEP use (Table 3).

Demographically, younger age was a key factor; MSM aged 18 to 28 years were 2.6 times more likely to engage in this behavior compared to those aged 40 and over (AOR: 2.59; 95% CI: 1.27–5.31). Regarding relationship context, having a steady partner emerged as one of the strongest predictors, increasing the odds of the outcome by more than fourfold (AOR: 4.57; 95% CI: 3.32–6.28). Sexual orientation was also a powerful determinant, with homosexual (AOR: 6.04), bisexual (AOR: 5.30), and pansexual (AOR: 8.67) participants showing significantly higher odds compared to the reference group.

Finally, behavioral and clinical histories were also significant predictors. The odds of the risk behavior nearly tripled for participants whose primary practice was receptive or insertive anal sex. Furthermore, a previous diagnosis of an STI also showed a strong and significant association with the outcome (AOR: 1.49; 95% CI: 1.11–1.99). Table 3 presents the full results of the regression model, including both crude and adjusted odds ratios.

**Figure 1:**
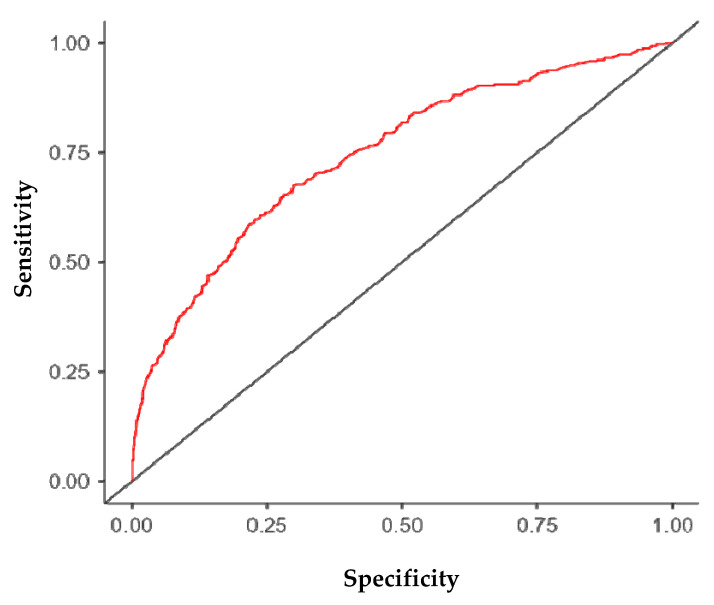
ROC curve of the adjusted model.

Additionally, the Receiver Operating Characteristic (ROC) curve, presented in Figure 1, demonstrates the quality of the model’s fit. The model’s accuracy, as measured by the area under the curve (AUC), was found to be 0.746, indicating acceptable discriminatory power. These results suggest that the model performed well in estimating the probability of engaging in unprotected anal sex without PrEP based on the included variables.

## 4. Discussion

Our study revealed concerning data regarding the practice of unprotected anal sex (without external condoms) and the absence of PrEP use among MSM in Brazil and its associated factors. A significant proportion of participants (69.4%) reported engaging in unprotected anal sex without PrEP in the past year. We identified factors associated with unprotected (insertive/active or receptive/passive) anal sex without PrEP among MSM: age between 18 and 28 years; homosexual, bisexual, or pansexual orientation; having a steady partner; engaging primarily in receptive or insertive anal sex; and having an STI diagnosis.

The rate of unprotected anal sex and the absence of PrEP use among MSM in Brazil is considerably higher than those observed in other international studies: 10% in Sweden [15]; 14% to 40% in the United States [16,17]; and 40% in Canada [18]. However, cultural and economic differences among these countries must be considered. Despite this, international evidence also supports, in part, the vulnerability profile identified in our study, highlighting the importance of targeted preventive interventions for this population [19].

Regarding age, our study found that MSM aged 18 to 28 years were more likely to engage in unprotected anal sex without PrEP compared to those aged 40 years and older. A Brazilian study found that 41.9% of MSM aged 18 to 24 engaged in unprotected anal sex [20]. Beyond Brazil, the association between younger MSM and unprotected anal sex has been identified in other countries, such as Spain [19]. It is noteworthy that the age-related data indicate a higher likelihood of risky sexual behavior in the studied group. Further research is needed to explore the relationship between age and the reasons behind engaging in high-risk sexual practices for HIV and other STI acquisition. Additionally, a high prevalence of HIV has been reported among adolescent and young MSM in Indonesia [21]. In Brazil, the reality of high HIV infection rates among adolescents is similar [7]. A Brazilian study also confirmed the high vulnerability of MSM to new HIV and STI diagnoses [22]. The findings related to age and unprotected sex suggest an urgent need for targeted interventions that provide clear and accessible information for younger groups.

Regarding sexual orientation, our results showed that homosexual, bisexual, and pansexual MSM were more likely to engage in unprotected anal sex without PrEP. Similar data were reported in a Brazilian study [20]. Our study identified a group of MSM, mostly identifying as homosexual, with sociodemographic and behavioral characteristics that may be related to HIV acquisition risk perception and high adherence to unprotected sex. However, our study did not investigate risk perception or adherence to combined prevention. Future studies should explore Brazilian MSM’s risk perception and preventive behavior adoption, including knowledge about PrEP and other prevention methods [4].

Regarding partner type, MSM with steady partners were more likely to engage in unprotected anal sex without PrEP compared to those without a steady partner. This finding may be related to trust within the sexual relationship. However, risks remain, and couples should adopt different prevention strategies, especially those in key populations. Additionally, international studies have reported divergent findings [23]. These discrepancies may be influenced by economic and cultural differences. For example, in lower-income countries, access to healthcare services—and consequently, adherence to multiple prevention strategies—is more challenging when compared to high-income countries. Furthermore, regarding culture and laws, in less progressive countries, sexual practices among MSM are considered illegal. As a result, this population faces disproportionate barriers to engaging in preventive practices when compared to populations in less conservative cultural contexts. In addition, a Spanish study supported our findings, showing that the risk of unprotected sex was higher among men in relationships [19].

Beyond individual behaviors, it is crucial to consider the psychosocial and structural factors that shape HIV vulnerability among MSM in Brazil. Stigma and discrimination remain pervasive in society and even within healthcare settings, potentially deterring MSM from seeking testing, prevention services, or disclosing their sexual practices to providers. This can create significant barriers to accessing and adhering to PrEP. Furthermore, regional inequalities in Brazil affect the availability of specialized health services and the distribution of prevention resources. Our findings, therefore, should be interpreted within this context, where individual choices are constrained by broader social and structural determinants that were not directly measured in our study but likely influence the observed behaviors.

Regarding the most frequent sexual practice, MSM who primarily engaged in receptive anal sex were more likely to practice unprotected anal sex without PrEP. Similarly, MSM who primarily engaged in insertive anal sex were more likely to have unprotected anal sex without PrEP compared to those with other predominant sexual practices. Despite these practices, some men may benefit from circumcision, which is more common in cultures different from Brazil’s. Evidence from a systematic review (including 21 observational studies with 71,693 participants) indicated that male circumcision could provide protection against HIV infection among MSM who primarily practice insertive anal sex [24], especially in low- and middle-income countries [25]. These findings suggest that in contexts where condoms and PrEP are not used, circumcision may offer some protection for MSM. High-risk behaviors for HIV and other STI acquisition have been reported in other contexts. For example, a large Asian study involving 10,413 MSM found that among the 7311 participants who engaged in receptive anal intercourse, 47.5% had unprotected receptive anal sex with internal ejaculation [26]. In the Brazilian context, a study comparing different anatomical sites of chlamydia and gonorrhea infection found a higher prevalence of these pathogens in anal samples, confirming that receptive sex may pose a greater risk for HIV and other STI acquisition [27].

Regarding STI diagnosis, MSM who had been diagnosed with an STI were more likely to engage in unprotected anal sex without PrEP compared to those without an STI diagnosis. Evidence indicates that younger MSM have higher STI diagnosis rates [28], which may indicate sexual risk behavior patterns, as evidenced by the high proportion of MSM in our study engaging in unprotected anal sex without PrEP.

Data for this study were collected between April and May 2020, during the initial and most restrictive phase of the COVID-19 pandemic in Brazil. This context may have influenced sexual behaviors and access to prevention methods. Lockdowns and social distancing measures could have altered sexual networking patterns, potentially reducing casual partnerships but increasing sexual activity within steady relationships, which our study identified as a risk factor. Concurrently, disruptions in healthcare services may have limited access to condoms, STI/HIV testing, and PrEP initiation or refills. While our study did not measure these effects directly, the pandemic context is an important consideration when interpreting the high prevalence of the risk behavior and represents a unique historical factor affecting this cohort.

This study presents limitations that should be considered for future research and result interpretation. Firstly, it is a cross-sectional design, which makes it impossible to establish causal relationships between the variables studied. Secondly, participants were recruited through social media and platforms specifically targeting MSM, which may have led to an overrepresentation of the population of interest, as these online spaces cater specifically to LGBTQIA+ audiences. Consequently, MSM without well-defined identities and sexual orientations may not have been adequately represented in the study.

## 5. Conclusions

This study reveals a high prevalence (69.4%) of condomless anal sex combined with non-use of PrEP among a large sample of Brazilian MSM, highlighting a critical gap in the effectiveness of combination prevention strategies. The key factors associated with this high-risk profile were younger age (18–28 years), having a steady partner, identifying with non-heterosexual orientations, and a history of STIs. These findings identify a clear profile of vulnerability and have significant public health implications. They indicate that prevention messages must be tailored to young MSM and challenge the common misconception that steady partnerships are inherently safe.

Health policies in Brazil must intensify efforts to integrate PrEP education and access into routine healthcare for MSM, addressing structural barriers and stigma. Future research should employ longitudinal designs to establish causality and qualitative methods to explore the psychosocial drivers behind these behaviors and, based on the results, propose personalized interventions tailored to the specific characteristics of the studied group. Addressing this vulnerability is essential for Brazil to achieve the UNAIDS 2030 goal of eliminating the HIV epidemic.

## Figures and Tables

**Table 1:** Sociodemographic and behavioral variables of men who have sex with men (n = 1357). Brazil, 2024.

Variable		N°	%
gender identity			
	Cisgender man	1195	88.1
	Transgender man	13	1.0
	Intergender	14	1.0
	Other	135	9.9
sexual orientation			
	Homosexual	1115	82.2
	Heterosexual	20	1.5
	Bisexual	167	12.3
	Pansexual	38	2.8
	Asexual	4	0.3
	Other	13	1.0
Age range			
	18 to 28 years	1008	73.3
	29 to 39 years	348	25.6
	40 years and over	1	0.1
Years of study			
	<11 years	266	19.6
	>11 years	1091	80.4
Skin Color			
	White	623	45.9
	Black	164	12.1
	Brown	530	39.1
	Yellow	18	1.3
	Did not declare	16	1.2
	Other	6	0.4
Work Situation			
	Formal work	648	47.8
	Informal work	183	13.5
	Unemployed	188	13.9
	Retired/pensioner	4	0.3
	Student	334	24.6
Marital status			
	Single	1132	83.0
	Married	58	4.3
	Stable union/partnership	160	11.8
	Divorced/separated	5	0.4
	Widower	2	0.1
Fixed Partner			
	Yes	540	39.8
	No	817	60.2
Use alcohol			
	Yes	1028	75.8
	No	329	24.2
Use Tobacco			
	Yes	254	18.7
	No	1103	81.3
Use other drugs			
	Yes	356	26.2
	No	1101	73.8
Most frequent sexual practice			
	Oral	347	25.6
	Receptive anal	482	35.5
	Insertive anal	435	32.1
	Likes to observe	11	0.8
	Other	82	6.0

**Table 2:** Characterization of men who have sex with men and the association of independent variables with the practice of unprotected anal sex without PrEP (n = 1357). Brazil, 2025.

Variables		Anal Intercourse Without Condom and Without PrEP in the Last 12 Months			
		Yes 942 (69.4%)	No 415 (30.6%)	Total 1357 (100%)	Pearson Chi-Square
Gender identity					0.02
	Cisgender	844 (70.6)	351 (29.4)	1195 (100)	
	Transgender	10 (76.9)	3 (23.1)	13 (100)	
	Intergender	10 (71.4)	4 (28.6)	14 (100)	
	Other	78 (57.8)	57 (42.2)	135 (100)	
Sexual orientation					<0.001
	Homosexual	798 (71.6)	317 (28.4)	1115 (100)	
	Heterosexual	4 (20.0)	16 (80.0)	20 (100)	
	Bisexual	108 (64.7)	59 (35.3)	167 (100)	
	Pansexual	27 (71.1)	11 (28.9)	38 (100)	
	Asexual	0 (0.0)	4 (100.0)	4 (100)	
	Other	5 (38.5)	8 (61.5)	13 (100)	
Skin color					0.127
	White	420 (67.4)	203 (32.6)	623 (100)	
	Black	124 (75.6)	40 (24.4)	164 (100)	
	Brown	374 (70.6)	156 (29.4)	530 (100)	
	Yellow	9 (50.0)	9 (50.0)	18 (100)	
	Did not declare	10 (62.5)	6 (37.5)	16 (100)	
	Other	5 (83.3)	1 (16.7)	6 (100)	
Work situation					0.077
	Formal Work	467 (72.1)	181 (27.9)	648 (100)	
	Informal Work	124 (67.8)	59 (32.2)	183 (100)	
	Unemployed	131 (69.7)	57 (30.3)	188 (100)	
	Retired or Pensioner	1 (25.0)	3 (75.0)	4 (100)	
	Student	219 (65.6)	115 (34.4)	334 (100)	
Marital status					0.002
	Single	764 (67.5)	368 (32.5)	1132 (100)	
	Married	39 (67.2)	19 (32.8)	58 (100)	
	stable union, Live together or Cohabiting	133 (83.1)	27 (16.9)	160 (100)	
	Divorced or Separated	4 (80.0)	1 (20.0)	5 (100)	
	Widower	2 (100)	0 (0.0)	2 (100)	
Fixed partner					
	Yes	447 (82.8)	93 (17.2)	540 (100)	<0.001
	No	495 (60.6)	322 (39.4)	817 (100)	
Alcohol use					
	Yes	728 (70.8)	300 (29.2)	1028 (100)	0.048
	No	214 (65.0)	115 (35.0)	329 (100)	
Tobacco use					
	Yes	180 (70.9)	74 (29.1)	254 (100)	0.578
	No	762 (69.1)	341 (30.9)	1103 (100)	
Drug use (Illicit)					
	Yes	262 (73.6)	94 (26.4)	356 (100)	0.046
	No	680 (67.9)	321 (32.1)	1001 (100)	
More frequent sexual practice					
	Oral	203 (58.5)	144 (41.5)	347 (100)	<0.001
	Receptive anal	359 (74.5)	123 (25.5)	482 (100)	
	Insertive anal	339 (77.9)	96 (22.1)	435 (100)	
	Observer	1 (9.1)	10 (90.9)	11 (100)	
	Other	40 (48.8)	42 (51.2)	82 (100)	
Used a condom in the last sexual intercourse					
	Yes	346 (51.3)	328 (48.7)	674 (100)	<0.001
	No	596 (87.3)	87 (12.7)	683 (100)	
Use lubricating gel during sexual intercourse					
	Yes	763 (69.4)	337 (30.6)	1100 (100)	0.929
	No	179 (69.6)	78 (30.4)	257 (100)	
HIV Test					
	Yes	776 (72.1)	300 (27.9)	1076 (100)	<0.001
	No	166 (59.1)	115 (40.9)	281 (100)	
Do you know of a place where you can do one? quick test HIV					
	Yes	689 (70.2)	292 (29.8)	981 (100)	0.292
	No	253 (67.3)	123 (32.7)	376 (100)	
Talked to any health professional about carrying out an HIV test					
	Yes	459 (70.4)	193 (29.6)	652 (100)	0.451
	No	483 (68.5)	222 (31.5)	705 (100)	
Talked to a friend about taking an HIV test					
	Yes	456 (70.6)	190 (29.4)	646 (100)	0.372
	No	486 (68.4)	225 (31.6)	711 (100)	
It received free male condom					
	Yes	563 (69.4)	248 (30.6)	811 (100)	0.998
	No	379 (69.4)	167 (30.6)	546 (100)	
Read information on internet about HIV prevention					
	Yes	788 (69.4)	348 (30.6)	1136 (100)	0.925
	No	154 (69.7)	67 (30.3)	221 (100)	
Read information in printed material about HIV prevention					
	Yes	479 (69.9)	206 (30.1)	685 (100)	0.681
	No	463 (68.9)	209 (31.1)	672 (100)	
STI diagnosis					
	Yes	356 (76.7)	108 (23.3)	464 (100)	<0.001
	No	586 (65.6)	307 (34.4)	893 (100)	
Do you know someone living with HIV?					
	Yes	549 (70.2)	233 (29.8)	782 (100)	0.463
	No	393 (68.3)	182 (31.7)	575 (100)	
Sex worker					
	Yes	11 (57.9)	8 (42.1)	19 (100)	0.272
	No	931 (69.6)	407 (30.4)	1338 (100)	
Age Range					
	18 to 28 years	709 (70.3)	299 (29.7)	1008 (100)	0.160
	29 a 39 years	233 (67.0)	115 (33.0)	348 (100)	
	40 years and over	0 (0.0)	1 (100)	1 (100)	
Years of Study					
	<11 years	175 (65.8)	91 (34.2)	266 (100)	0.152
	>11 years	767 (70.3)	324 (29.7)	1091 (100)	
Multiple Partners					
	Yes	450 (65.6)	236 (34.4)	686 (100)	0.002
	No	492 (73.3)	179 (26.7)	671 (100)	

**Table 3:** Adjusted model of factors associated with unprotected anal sex and lack of pre-exposure prophylaxis (PrEP) use among Brazilian men who have sex with men (n = 1357). Brazil, 2025.

Variables		Crude OR [95% IC *]	*p*	Adjusted OR [95% IC *]	*p*
Age Range					
	18 a 28 years	2.12 [1.08–4.12]	0.028	2.59 [1.27–5.31]	0.009
	29 a 39 years	1.81 [0.86–3.76]	0.114	1.51 [0.69–3.31]	0.301
	40 years and more	Ref.	Ref.	Ref.	Ref.
Sexual Orientation:					
	Homosexual	6.04 [2.11–17.29]	<0.001	6.04 [1.80–20.20]	0.004
	Heterosexual	0.60 [0.13–2.72]	0.508	0.85 [0.15–4.61]	0.852
	Pansexual	4.39 [1.47–13.07]	0.008	5.30 [1.53–18.41]	0.009
	Bisexual	5.89 [1.67–20.70]	0.006	8.67 [2.07–36.30]	0.003
	Other	Ref.	Ref.	Ref.	Ref.
Gender					
	Cisgender	1.7 [1.22–2.53]	<0.001	1.29 [0.83–2.01]	0.245
	Transgender	2.4 [0.64–9.25]	0.19	3.09 [0.68–13.97]	0.142
	Intergender	1.8 [0.5–6.12]	0.32	1.24 [0.34–4.52]	0.738
	Other	Ref.	Ref.	Ref.	Ref.
Skin color					
	White	0.414 [0.04–3.57]	0.422	0.17 [0.01–1.72]	0.136
	Black	0.620 [0.07–5.47]	0.667	0.27 [0.02–2.82]	0.280
	Brown	0.479 [0.05–4.14]	0.504	0.21 [0.02–2.13]	0.191
	Yellow	0.200 [0.01–2.07]	0.177	0.07 [0.006–0.95]	0.046
	Not declared	0.333 [0.03–3.58]	0.364	0.17 [0.01–2.33]	0.187
	Other	Ref.	Ref.	Ref.	Ref.
Fixed Partner					
	Yes	3.13 [2.40–4.07]	0.001	4.57 [3.32–6.28]	0.001
	No	Ref.	Ref.	Ref.	Ref.
Alcohol use					
	Yes	1.30 [1.0–1.70]	0.048	1.07 [0.79–1.46]	0.633
	No	Ref.	Ref.	Ref.	Ref.
Use of illicit drugs					
	Yes	1.32 [1.0–172]	0.047	1.10 [0.81–1.50]	0.510
	No	Ref.	Ref.	Ref.	Ref.
Most frequent sexual practice					
	Oral	1.480 [0.91–2.39]	0.111	1.41 [0.80–2.47]	0.231
	Receptive anal	3.065 [1.89–4.94]	0.001	2.73 [1.57–4.75]	0.001
	Insertive anal	3.708 [2.27–6.04]	0.001	2.80 [1.59–4.92]	0.001
	Observer	0.105 [0.01–0.85]	0.035	0.10 [0.010–1.09]	0.059
	Other	Ref.	Ref.	Ref.	Ref.
HIV Test					
	Yes	1.79 [1.36–2.35]	0.001	1.35 [0.97–1.87]	0.073
	No	Ref.	Ref.	Ref.	Ref.
STI diagnosis					
	Yes	1.73 [1.34–2.23]	0.001	1.49 [1.11–1.99]	0.008
	No	Ref.	Ref.	Ref.	Ref.
Years of Study					
	<11 years	0.812 [0.61–1.08]	0.152	1.22 [0.86–1.72]	0.260
	>11 years	Ref.	Ref.	Ref.	Ref.
Multiple Partners					
	Years	0.694 [0.55–0.87]	0.002	0.49 [0.37–0.66]	0.001
	No	Ref.	Ref.	Ref.	Ref.

* Confidence Interval.

## Data Availability

The original contributions presented in this study are included in the article. Further inquiries can be directed to the corresponding authors.

## Supplementary Materials

The following supporting information can be downloaded at: https://www.mdpi.com/article/10.3390/idr17060149/s1, S1: Questionnaire: Sociodemographic and Behavioral Data Questionnaire for Men Who Have Sex with Men.
