# Supplementary material for: Factors Associated with Condomless Anal Sex and Absence of Pre-Exposure Prophylaxis (PrEP) Use Among Brazilian Men Who Have Sex with Men: A Cross-Sectional Study
Source: Infect Dis Rep. 2025 Dec 12;17(6):149. doi: 10.3390/idr17060149 (PMC12732578; doi:10.3390/idr17060149)
Supplement: Supplementary file 1 [file idr-17-00149-s001.zip › idr-3970264-supplementary.pdf]

Supplementary Materials S1 - Questionnaire

Sociodemographic and Behavioral Data Questionnaire for Men Who Have Sex with Men

1 – What is your gender identity?
a) Cis man
b) Trans man
c) Intergender
d) Other: (specify) ________

2 – What is your sexual orientation?
a – Homosexual
b – Heterosexual
c – Bisexual
d – Pansexual
e – Asexual
f – Other: (specify) ______

3 – How old are you? (Complete years)
(Options from 18 to 80 years old)

4 – In which state of Brazil do you live?

5 – What color is your skin?
1. ( )White 4.( )Yellow
2. ( )Black 5.( )Indigenous
3. ( )Brown 6.( ) Other:

6 – How many years of study do you have? (complete years of study)
(Options from 1 to 30 years of study)

7 – What is your current work situation:
1. ( )Not Applicable 6.( )benefit
2. ( ) signed contract 7. ( ) Unemployed
3. ( )Informal work 8.( )Professional Sex
4. ( )self-employed 9.( )Others:__________
5. ( )retired

8 – What is your monthly family income
(Options in minimum wages)

9. What is your current Marital Status?
1. ( ) Single, 2 ( ) Married/Living together/Consensual union/Cohabiting,
3 ( ) Divorced/Separated, 4 ( ) Widowed.

10. Do you have a steady sexual partner?
( ) Yes No

11. If you have a steady partner, do you live together?<br>1 ( ) Yes 2. ( ) No
12. You use alcohol<br>(Alcoholic beverage)?<br>1 ( ) Yes, 2 ( ) No
13. If yes, how often:<br>1 ( ) Daily, 2 ( ) Weekly,<br>3 ( ) Monthly,4. ( ) Sporadically
14. Do you use tobacco (paper or straw cigarettes)? :<br>1 ( ) Yes, 2 ( ) No
15. Do you have an active sex life?<br>( ) 1 Yes ( ) 2 No
16. If yes, what is the number of partners in the last 3 months?
17. What is the number of sexual partners in the last year?
18. Do you have multiple sexual partners?<br>( ) 1 Yes ( ) 2 No
19. At what age did your sexual life begin?
frequent sexual practice ?<br>1. Oral ( )<br>2. Receptive anal ( )<br>3. Insertive anal ( )<br>4. Other _______
21. Have you had any anal sex without a condom in the last year (passive and/or insertive without a condom)?<br><br>( ) Yes No
22. Use a male condom in all sexual relations<br>( ) Yes No
23. Did you use a male condom during your last sexual encounter?<br>( ) Yes No
24. Using lubricating gel during sexual intercourse<br>(__) Yes No
25. Have you ever tested yourself for HIV in your life?<br>( ) Yes No

26. If you were tested, what was the result of the test?<br>( ) Positive negative
27. Do you know of any place where I can take an HIV test?<br>( ) Yes No
28. Have you received advice from a healthcare professional about HIV testing?<br>( ) Yes No
29. Have you spoken to a friend who advised you about testing for HIV?<br>( ) Yes No
30. Have you received free male condoms in the last 12 months?<br>( ) Yes No
31. Have you read information on the internet about HIV prevention in the last 12 months?<br>( ) Yes No
32. Have you read information in printed materials about prevention in the last 12 months?<br>( ) Yes No
33. Have you ever had a positive diagnosis of any Sexually Transmitted Infection?<br>( ) Yes No
34. Do you know anyone living with HIV?<br>( ) Yes No
35. Are you a Sex Worker?<br>( ) Yes No
36. Do you use Pre-Exposure Prophylaxis?<br>( ) Yes No
